# Practical Medicine

**Published:** 1864-05

**Authors:** 


Practical Medicine.—*On the Hypodermical Treatment of Disease.*—In 1858–59 papers were communicated to some of the Medical Journals by Charles Hunter, Esq., M. R. C. S., establishing the application of "the puncture" to the treatment of diseases affecting the organism generally, or at points far remote from the point of Medicinal introduction.

This method has special value in subduing cerebral and spinal excitement.

The alkaloids of belladonna, aconite, &c., were first employed hypodermically by Mr. Hunter, although the bulk of his observations were relative to the action of morphia. Though the value of this mode of treatment is most marked in affections of the nervous system, from the rapid way in which it will produce sleep, and lull, or cure pain, still there are many other affections—blood diseases—which show the superiority this method has over others in checking disease.

Four or five grains of quinine injected beneath the skin, are equal to five or six times that quantity taken by the stomach. Small doses of morphine, given in this way, will procure sleep in delirium tremens, when large stomachic doses fail.

The solutions used are the most concentrated that can be produced. The less the bulk of the fluid injected the better. Three minims thrown in at one place, produces no pain.

The punctures should not be made close together, or accute inflammation of the cellular tissue will result.

The fluid employed should be as near neutral as possible.

The mode of action of our narcotics and sedatives, is so little known or thought about, that practitioners are often in doubt as to which agent to employ in such and such a case. The special parts of the nervous system upon which the various alkaloids act, are not sufficiently considered.

By the hypodermical administration of the medicines, these different effects are better seen, than when given by the stomach.

When a dose is given by the mouth, it has to pass into the intestinal tract, and through the portal circulation before it reaches the heart, and its systematic effects are more slowly developed. But when introduced into the cellular tissue, the absorbent vessels carry it at once to the fountain head of arterial supply, its effects are more powerful, and better observed.

The effects of morphia and atropine on the same subject, are thus described:

John A——, with sciatica of some years standing, was injected in the arm with half grain of morphia. The pulse at the time was 80, quiet and small; in one minute it was 76, fuller and stronger in quality; in twelve minutes, the quality remained full, but in rate diminished to 66; the brain circulation was influenced, he was drowsy; he slept better that night than for months before.

If his pulse is light, and the patient excited, the action of the heart is diminished in proportion to the dose injected. Thus in mania, it is reduced from 120 to 80, in four minutes, and the respirations diminished accordingly, at the same time the cutaneous action is increased.—*Lancet.*

